# The Glycogen Synthase Kinase-3β Inhibitor LSN 2105786 Promotes Zebrafish Fin Regeneration

**DOI:** 10.3390/biomedicines7020030

**Published:** 2019-04-19

**Authors:** Swapnalee Sarmah, Courtney Curtis, Jennifer Mahin, Mark Farrell, Thomas A. Engler, Manuel V. Sanchez-Felix, Masahiko Sato, Yanfai Linda Ma, Shaoyou Chu, James A. Marrs

**Affiliations:** 1 Department of Biology, Indiana University-Purdue University Indianapolis, Indianapolis, IN 46202, USA; ssarmah@iupui.edu (S.S.); clcurtis@iupui.edu (C.C.); jmahin@iupui.edu (J.M.); mfarrell@iupui.edu (M.F.); 2 Lilly Research Laboratories, Indianapolis, IN 46225, USA; engler_thomas@lilly.com (T.A.E.); Manuel.sanchez-felix@novartis.com (M.V.S.-F.); msato@iupui.edu (M.S.); ma_linda@lilly.com (Y.L.M.); syc1321@gmail.com (S.C.); 3 Novartis Institute for BioMedical Research, Cambridge, MA 02139, USA; 4 Molecular Templates, Austin, TX 78729, USA

**Keywords:** zebrafish, glycogen synthase kinase-3β, bone healing, regeneration, caudal fin

## Abstract

The Wnt pathway has been shown to regulate bone homeostasis and to influence some bone disease states. We utilized a zebrafish model system to study the effects of a synthetic, orally bioavailable glycogen synthase kinase-3β (GSK3β) inhibitor LSN 2105786, which activates Wnt signaling during bone healing and embryogenesis. GSK3β inhibitor treatment was used to phenocopy GSK3β morpholino oligonucleotide (MO) knockdown in zebrafish embryos. Human and zebrafish synthetic mRNA injection were similarly effective at rescue of GSK3β MO knockdown. During caudal fin regeneration, bony rays are the first structure to differentiate in zebrafish fins, providing a useful model to study bone healing. Caudal fin regeneration experiments were conducted using various concentrations of a GSK3β inhibitor, examining duration and concentration dependence on regenerative outgrowth. Experiments revealed continuous low concentration (4–5 nM) treatment to be more effective at increasing regeneration than intermittent dosing. Higher concentrations inhibited fin growth, perhaps by excessive stimulation of differentiation programs. Increased Wnt responsive gene expression and differentiation were observed in response to GSK3b inhibitor treatment. Activating Wnt signaling also increased cell proliferation and osteoblast differentiation in fin regenerates. Together, these data indicate that bone healing in zebrafish fin regeneration was improved by activating Wnt signaling using GSK3b inhibitor treatment. In addition, caudal fin regeneration is useful to evaluate dose-dependent pharmacological efficacy in bone healing, various dosing regimens and possible toxicological effects of compounds.

## 1. Introduction

The Wnt signaling pathway controls bone formation, homeostasis, and repair [1]. Wnt ligands are cysteine-rich, secreted glycoproteins that regulate embryonic development by controlling cell growth, differentiation, and apoptosis [2]. In bone growth, canonical Wnt, β-catenin-dependent signaling is activated when the Wnt ligand binds to a transmembrane Frizzled (Fz) receptor with the co-receptors low density lipoprotein receptor-related proteins 5/6 (LRP 5/6). Receptor engagement activates Disheveled (Dsh) family proteins, which in turn, disrupts the β-catenin destruction complex, preventing cytoplasmic glycogen synthase kinase-3β (GSK3β) from phosphorylating β-catenin. In the absence of Wnt signaling, the GSK3β serine-threonine kinase targets β-catenin N-terminal sequences, and phosphorylation leads to polyubiquitin modification that marks β-catenin for degradation [3]. Wnt signaling disrupts GSK3β phosphorylation of β-catenin, and unphosphorylated β-catenin accumulates in the cytoplasm and translocates to the nucleus. Nuclear β-catenin displaces histone deacetylase (HDAC) corepressors from T-cell factor/Lymphoid enhancer factor (TCF/LEF), recruits histone acetylases (HAT) and activates Wnt target genes. Dickkopfs (Dkks), sclerostin (SOST) and secreted Frizzled-related proteins (SFRPs) antagonize the Wnt/β-catenin signaling pathway [4].

Loss of function mutations in the LRP 5/6 co-receptor produce osteoporosis-pseudoglioma syndrome, where patients have significantly lower bone mass and an increased risk of fracture [5]. Conversely, gain-of-function mutations that prevent binding of the Wnt antagonist Dkk to the LRP 5/6 co-receptor produce a high bone mass phenotype with no other obvious defects [6]. These observations show that Wnt signaling is a critical regulator of bone formation. Beta-catenin signaling plays a crucial role in bone formation by regulating osteoblast differentiation and suppressing osteoclast activity [7,8]. Additionally, β-catenin is necessary for mesenchymal progenitors to differentiate into osteoblasts [9,10]. Ectopic expression of Wnt14, a canonical Wnt signaling pathway ligand, in early mouse embryos enhanced osteogenesis and expansion of osteoblast markers such as Runx2, osterix and osteocalcin [11]. Wnt10b, another canonical Wnt signaling pathway ligand, stimulates osteoblastogenesis (Runx2 and osterix expression) and inhibits adipogenesis [12].

The canonical Wnt pathway is important in the bone healing process [13]. Wnt pathway markers, including Wnt ligands, were upregulated during the early stages of fracture repair, and β-catenin levels were elevated throughout fracture repair before declining to basal levels as healing completes [13]. Previous studies showed Dkk1 inhibited Wnt signaling during the first week following fracture blocks healing, leaving undifferentiated mesenchymal tissue in place of a callus at the site of injury [14]. Antibodies to Dkk1 and sclerostin were shown to powerfully enhance bone anabolism and facilitate fracture repair in animal models [15,16,17]. Additionally, parathyroid hormone (PTH), which also regulates the Wnt signaling pathway [18] was shown to enhance healing in animal and clinical studies [19,20,21].

Wnt/β-catenin signaling is the earliest genetically identified step in fin regeneration and is required during all stages of regeneration [22]. The Wnt ligand, Wnt10a, which signals through the canonical Wnt pathway, is upregulated during the first few hours following amputation of zebrafish fin (hours post-amputation, hpa) [22]. At the same time, β-catenin expression is detectable in the cells of the wound epidermis, where it is maintained throughout regeneration. Additionally, Wnt signaling transcriptional target *lef1* is expressed in the wound epidermis as early as 12 hpa in zebrafish [23]. Previous studies showed that inhibition of fibroblast growth factor (FGF) signaling does not affect expression of *lef1*, and β-catenin signaling is sufficient to repress expression of FGF target genes [24]. These results show that β-catenin signaling is upstream of FGF, Shh and Bmp2b signaling, directing blastema cells to produce osteoblasts [18,22].

In addition to the regenerative response, β-catenin signaling is also needed to establish the blastema. Using a transgenic zebrafish TOPdGFP, β-catenin signaling is detected in the blastema by 48 hpa. Additionally, Wnt signaling target genes (*axin2* and *sp8*) expression is activated during blastema formation [25]. The β-catenin and FGF signaling are required for blastema establishment, and disrupting FGF receptor 1 (Fgfr1, a Wnt target) prevents the activation of cellular proliferation markers and blocks blastema formation [23].

Heat shock inducible Dkk (Wnt signaling inhibitor) transgenic zebrafish were used to block β-catenin signaling at various times post amputation and showed that β-catenin signaling is needed during each stage of regeneration. Inhibition of β-catenin signaling at 0 or 1 day post-amputation (dpa) does not affect epidermal wound healing, but prevents blastema formation and subsequent regenerative outgrowth. Disrupting β-catenin signaling after the blastema has already formed allows only partial fin regeneration. Canonical Wnt-induced β-catenin signaling also regulates blastema maintenance during later outgrowth phases. Increasing β-catenin signaling enhanced fin regeneration rate. Fish with a mutation in one copy of the Wnt/β-catenin pathway inhibitor, *axin1*, display increased regenerative outgrowth at 7 dpa [25].

Interestingly, β-catenin independent, non-canonical Wnt signaling regulates fin regeneration. Blocking the non-canonical Wnt signaling pathway increased fin regenerative outgrowth, and over activation of non-canonical Wnt signaling completely blocks fin regeneration [25], showing that non-canonical Wnt signaling negatively feeds back on canonical/β-catenin signaling regulating normal regeneration.

Synthetic orally bioavailable GSK3β inhibitors, which also activate the canonical Wnt signaling pathway have been shown to enhance bone growth in rats [26]. In this study, we evaluated the ability of LSN 2105786 to enhance bone healing. The zebrafish caudal fin regeneration model was useful to evaluate dose-dependent pharmacological efficacy in bone healing, dosing regimens and possible toxicological effects. Experiments showed that a GSK3β inhibitor promoted caudal fin regeneration, and this model was useful to dissect the cellular and molecular mechanisms of GSK3β-inhibitor induced bone growth.

## 2. Experimental Section

### 2.1. Zebrafish Husbandry

Zebrafish (*Danio rerio*) AB strain and *Tg(TOP:GFP)* [27] transgenic line were raised and housed under standard laboratory conditions [28]. Indiana University-Purdue University Indianapolis School of Science Institutional Animal Care and Use Committee (IACUC) approved animal care and use protocol for this work (SC212R, 5/20/2015). Zebrafish, 6–12 months of age, were obtained from EKKWill Waterlife Resources (Ruskin, FL, USA) for caudal fin regeneration experiments and housed under standard laboratory conditions [28].

### 2.2. Adult Fin Amputation Assay

Zebrafish, 6–12 months of age, were used for caudal fin regeneration experiments. Fish were anesthetized in tricaine (Ethyl 3-aminobenzoate methanesulfonate), and approximately 50% of the caudal fin was amputated using a razor blade. Fish were placed in 2 L of water with various concentrations of GSK3β inhibitor compound (LSN 2105786) or dimethyl sulfoxide (DMSO; at least 1:1000 dilution) vehicle control and kept at 31 °C to promote rapid regeneration. Tank water was replaced daily, including fresh compound. Tanks and fish were rinsed between treatments to remove any residual compound.

At 4 and 7 dpa, fish were anesthetized and images of regenerating fins were collected using a Leica MZ12 microscope equipped with Leica DFC290 camera (Leica Microsystems Inc., Buffalo Grove, IL, USA). The length of the regenerate (from the amputation plane to the distal tip of the fin) at the third, fourth and fifth dorsal and ventral fin rays were measured using ImageJ software (NIH, http://rsb.info.nih.gov/ij/), and the average length of the regenerate was calculated for each fin.

### 2.3. Embryo Treatment with GSK3β Inhibitor

Embryos were incubated with LSN 2105786 (or vehicle controls) in embryo medium. Various concentrations of the drug were used to treat embryos from 6 to 28 h post fertilization (hpf), in Petri dishes wrapped with parafilm and maintained at 28.5 °C. Embryos were imaged using a Leica MZ12 microscope equipped with Leica DFC290 camera.

### 2.4. Whole Mount in situ Hybridization

Fins collected at various time points after amputation were fixed overnight at 4 °C in freshly made 4% paraformaldehyde in phosphate-buffered saline (PBS). Fins were then washed two times in PBS, dehydrated in methanol and stored at 20 °C overnight. Fins were rehydrated stepwise in methanol in PBS containing 0.1% Tween 20 (PBST). Next, fins were treated for 30 min in proteinase K (10 μg/mL) in PBST. Then, fins were washed two times in PBST, and post-fixed in 4% paraformaldehyde in PBS for 20 min. Fins were washed five times in PBST, and then prehybridized for 2 h at 70 °C in hybridization buffer (50% formamide, 5× SSC, 0.1% Tween 20, 50 μg/mL heparin, and 500 μg/mL yeast RNA). Following prehybridization, fins were hybridized overnight in hybridization buffer containing 0.5 μg/mL digoxigenin-labeled RNA probe at 70 °C. Then, fins were washed at 70 °C for 10 min each in 75% hybridization buffer/25% 2× SSC, 50% hybridization buffer/50% 2× SSC, 25% hybridization buffer/75% 2× SSC, and 2× SSC. Next, fins were washed two times in 0.05× SSC for 30 min each at 70 °C, followed by washes at room temperature for 5 min each in 75% 0.05× SSC/25% PBT, 50% 0.05× SSC/50% PBT, 25% 0.05× SSC/75% PBST, and PBST. Tissues were blocked for 2 h at room temperature in block buffer (2 mg/mL bovine serum albumin in PBST). Fins were then incubated overnight in a 1:5000 dilution of anti-digoxigenin antibody (Roche Diagnostics, Indianapolis, IN, USA) coupled to alkaline phosphatase in block buffer. The following day, fins were washed with PBST five times for 15 min each. Fins were then washed with alkaline phosphatase buffer (NTMT) three times for 15 min each, and then, fins were incubated with NBT/BCIP substrate in NTMT to allow color development. Development of the staining reaction was monitored carefully, and compound-treated and control fins were stopped at the same time to allow accurate comparison. Following color development, fins were washed five times in PBST and stored in 80% glycerol in PBST. Digoxigenin-labeled riboprobes for *shh*, *lef1*, *bmp2b*, *bmp4* and *sox9b* were synthesized using DIG RNA Labeling Kit (Roche, Indianapolis, IN, USA) according to manufacturer’s instructions. Images were collected using a Leica MZ12 microscope equipped with Leica DFC290 camera.

### 2.5. Whole Mount Immunostaining and Image Analysis

Fins were collected at various time points post-amputation and fixed overnight at 4 °C in 4% paraformaldehyde in PBS. Following fixation, fins were briefly washed several times with PBS. Fins were incubated overnight at room temperature in block solution, and then, fins were incubated with primary antibody in block solution for 1 to 3 days. Primary antibodies used were: mouse anti-Zns5 (Zebrafish International Resource Center, Eugene, OR, USA) at 1:200; and rabbit anti-phospho-histone H3 (Chemicon International, Inc.) at 1:500. After incubation in primary antibody, fins were washed with PBST six times for 10 min each, and then incubated overnight with secondary antibody in block solution. Goat anti-mouse Texas Red (Invitrogen), and goat anti-rabbit Alexa 488 (Invitrogen) were used. Nuclei were visualized by incubating fins in 1 µM To-PRO3 iodide for 1 h at room temperature. Confocal images were acquired using a Zeiss Observer Z1 LSM 700 confocal microscope (40× 1.1 NA W or 20× 0.8 NA objectives; Carl Zeiss Microscopy, Thornwood, NY USA). To measure cell proliferation phospho-histone 3 (PH3)-positive cells were counted from at least three fins per group. The number of PH3-positive cells per unit area was calculated for each fin using Volocity software (version 6.0.1, Perkin Elmer, Waltham, MA, USA).

### 2.6. DNA Sequence Alignment and 3D Protein Structure Prediction

DNA sequence alignment was done by using Clustal Omega program, The European Bioinformatics Institute (EMBL-EBI), Hinxton, Cambridgeshire, UK. 3D-protein structure prediction was done by Phyre2 web portal for protein modeling, prediction and analysis (Structural Bioinformatics Group, Imperial College, London, UK) [29].

### 2.7. Knockdown of gsk3β by Morpholino and Injection of Zebrafish gsk3β or Human GSK3β mRNA

Morpholino oligonucleotide (MO) was synthesized by Gene Tools, LLC (Philomath, OR, USA) to block the translation of *gsk3β* (this morpholino targets both *gsk3βa* and *gsk3βb*). The previously published sequence of the MO is GTTCTGGGCCGACCGGACATTTTTC [30].

MO concentration was determined spectrophotometrically. Different concentrations (2–6 ng/nL) of the MO were injected into 2–4 cell stage embryos to determine optimal effective concentrations. Injection of 1 nL of 3.5 ng/nL MO consistently produced defective embryos.

Zebrafish *gsk3β* resistant to MO and human *GSK3β* genes were synthesized in vitro at Lilly Research Laboratories and cloned in pAN104b and pcDNA3.1 vectors, respectively. Messenger RNAs were synthesized using T7 polymerase. Different concentrations of mRNA ranging from 100 to 500 ng/nL were injected along with the MO into 2–4 cell stage embryos. Co-injection of 200 ng/nL of either zebrafish or human synthetic GSK3β mRNA reduced the severity with MO (3.5 ng/nL) induced defects.

### 2.8. Statistical Analysis

Analyses on fin regenerate length and cell proliferation were performed using unpaired two-tailed Student’s *t*-test (GraphPad Software, La Jolla, CA, USA).

## 3. Results

### 3.1. Human GSK3β is Structurally and Functionally Equivalent to Zebrafish Gsk3β

**Figure 1:**
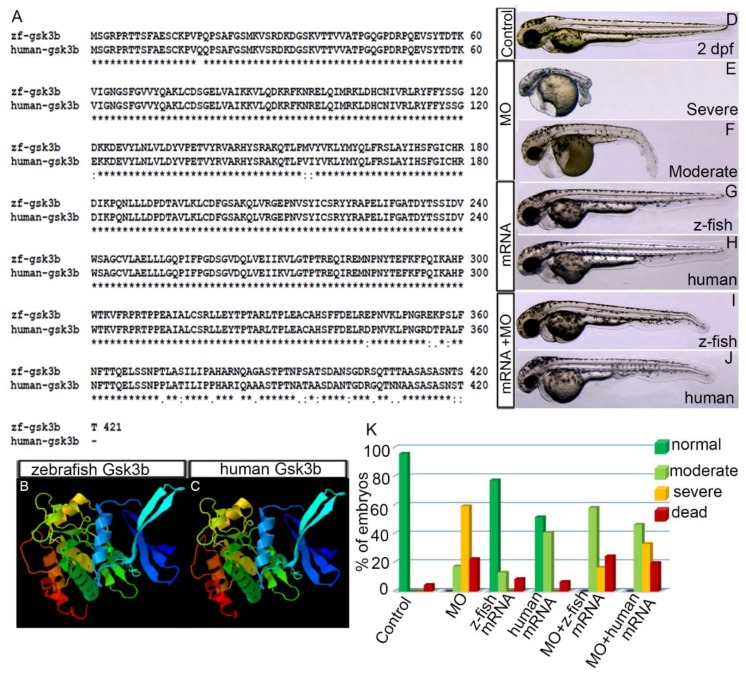
Zebrafish Gsk3β is structurally and functionally equivalent to human GSK3Β. (**A**) Alignment of protein sequences of zebrafish and human gsk3β. Identical residue (*), conserved (:), and semi conserved (.) substitutions are marked. (**B**,**C**) 3D-protein structure of zebrafish (**B**) and human (**C**) gsk3β. (**D**–**J**) Live image of the 2 dpf control embryo (**D**). (**E**,**F**) Gsk3β translation blocking morpholino oligonucleotide (MO) injection (3.5 ng/µL) produced severe and moderate defects. Live images of the severely (**E**) and moderately (**F**) defective morphant embryos. (**G**,**H**) Live images of the embryos injected with zebrafish Gsk3β and human GSK3β mRNA. (**I**,**J**) Live images of the embryos co-injected with either MO or zebrafish Gsk3β mRNA or MO and human GSK3β mRNA. (**K**) Graph showing the percentage of phenotypes of the embryos in different conditions: MO alone, zebrafish Gsk3β mRNA alone, human GSK3β mRNA, MO + zebrafish Gsk3β mRNA, MO + human GSK3β mRNA.

The human GSK3Β protein consisting of 420 amino acids is 95% identical and 98% similar to the zebrafish GSK3β protein consisting of 421 amino acids (Gsk3βa; Gsk3βb is 419 amino acids; Figure 1A). 3D-protein structure analyses predicted highly similar protein structures for zebrafish Gsk3β and human GSK3Β proteins (Figure 1B,C). To test whether human GSK3β is functionally equivalent to zebrafish Gsk3β, endogenous Gsk3β was knocked down in zebrafish embryos by injecting Gsk3β translation blocking morpholino (MO; 3.5 ng/nL) [30], and synthetic mRNA (200 ng/nL) encoding either zebrafish Gsk3β or human GSK3β protein was injected into the embryos at 2–4 cell stage. Embryos were examined at 1 and 2 days postfertilization (dpf). MO injected embryos were categorized into two phenotypic groups, severe and moderate, depending on the severity of the defects that include short bent body, small eye, pericardial edema (PE), brain ventricle edema and curly blistered tail (Figure 1E,F,K). Co-injection of the same amount of zebrafish or human synthetic GSK3β mRNA (200 ng/nL) with MO (3.5 ng/nL) reduced the severity of the defects, produced higher percentage of embryos with mild PE and with only slightly bent tails (Figure 1I,J). Zebrafish or human mRNA (200 ng/nL) injection alone produced only a few embryos with mild PE (Figure 1G,H). This experiment showed that injection of human synthetic GSK3β mRNA into zebrafish embryos rescued the zebrafish Gsk3β knockdown phenotype.

### 3.2. LSN 2105786 Treatment Upregulates Wnt Signaling in Zebrafish and Produces Eyeless Embryos

**Figure 2:**
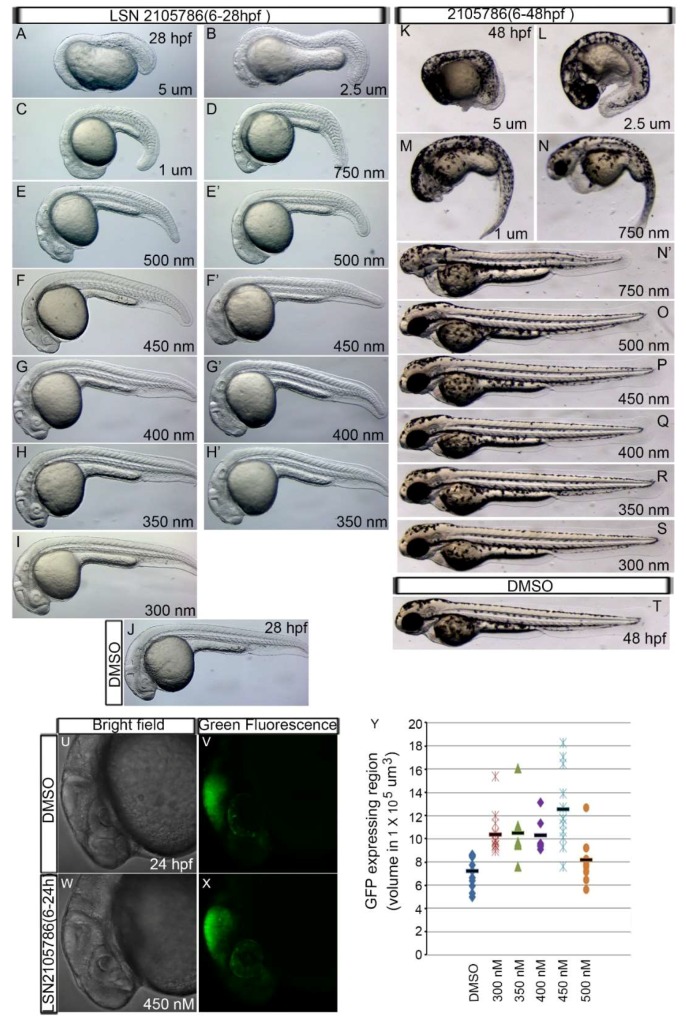
LSN 2105786 upregulates Wnt Signaling and produces eyeless phenotype. (**A**–**I**) Embryos treated with LSN 2105786 displayed the absent or small eye phenotype in a dose dependent manner characteristic of over-activated Wnt signaling at 28 hpf. (**J**) Live image of DMSO treated embryo showing normal eye at 28 hpf. (**K**–**S**) Embryos treated with LSN 2105786 displayed the absent or small eye phenotype in a dose dependent manner characteristic of over-activated Wnt signaling at 48 hpf. Panels E’, F’, G’, N’ are second examples of each concentration. (**T**) Live image of DMSO treated embryo showing normal eye at 48 hpf. (**U**–**X**) Bright field (**U**,**W**) and green fluorescence (**V**,**X**) images of the midbrain-hindbrain boundary region of *Tg*(*TOP:GFP*) embryos treated with DMSO (**U**,**V**) or 450 nM LSN 2105786 (**W**,**X**) at 24 hpf. (**Y**) Graph showing the GFP expressing midbrain-hindbrain boundary region of the embryos treated with different concentrations of LSN 2105786. Compared to DMSO, increased GFP volume in 300–450 nM was statistically significant (*p* < 0.001), and 500 nM effect was not statistically significant.

It was previously shown that over-activation of Wnt signaling after the mid-blastula transition produces small eye or eyeless phenotype [31]. To test whether LSN 2105786 acts upon the target enzyme GSK3β in zebrafish and upregulates Wnt signaling, embryos were treated with various concentrations (300 nM–5 µM) of the compound from 6 to 48 h post-fertilization (hpf). Treated embryos were examined at 1 and 2 dpf. LSN 2105786 treatment produced eye and other defects in zebrafish embryos, in a dose dependent manner, similar to the defects caused by over-activation of Wnt signaling. The defect was first observed at 1 dpf, which was more obvious at 2 dpf. Treatment with higher concentrations (750 nM–5 μM) of LSN 2105786 resulted in 100% eyeless embryos (*n* = 60 (three replicates); n, no. of embryos). The lower concentrations used (350–500 nM) resulted in variable phenotypes, ranging from reduced eye size to eyeless phenotypes. Treatment with 300 nM LSN 2105786 produced nearly normal appearance to eyes (92% near normal, 8% small eye; *n* = 66 (three replicates) (Figure 2A–T). To directly measure LSN 2105786 effects on a Wnt responsive reporter gene, TOP:GFP zebrafish transgenic strain embryos, which carries β-catenin-Tcf/Lef promoter binding sites that drive GFP gene expression [27], were treated with 350–500 nM compound from 6 to 28 hpf. Midbrain-hindbrain boundary region that expresses GFP was imaged at 28 hpf, and GFP intensity was analyzed using confocal microscopy. LSN 2105786 treated embryos showed increased GFP expression, and highest intensity was exhibited in the 450 nM compound treated embryos (Figure 2U–Y). GFP intensity decreased in 500 nM LSN 2105786 treated embryos (Figure 2Y), perhaps due to attenuation of Wnt signaling due to over-activation.

### 3.3. LSN 2105786 Continuous Treatment after Fin Amputation Accelerates Fin Regeneration

To test the effects of LSN 2105786 on the rate of zebrafish fin regeneration, half of the caudal fin was amputated from adult zebrafish (6 to 12 months old) and the fish were treated either with compound or with vehicle DMSO immediately following amputation. To ascertain the optimal treatment regimen for maximal regenerative outgrowth, fish were either treated intermittently (1, 2, 4, 6 and 8 hr/day) with higher concentrations (50–300 nM) or continuously with lower concentrations (1–50 nM) of LSN 2105786. Continuous exposure with high concentration of LSN 2105786 killed the fish. At 4 and 7 dpa, fins were imaged, and regenerative outgrowth was measured. Continuous exposures with low concentrations of LSN 2105786 were more effective at stimulating regeneration than intermittent exposure, as detailed below.

**Figure 3:**
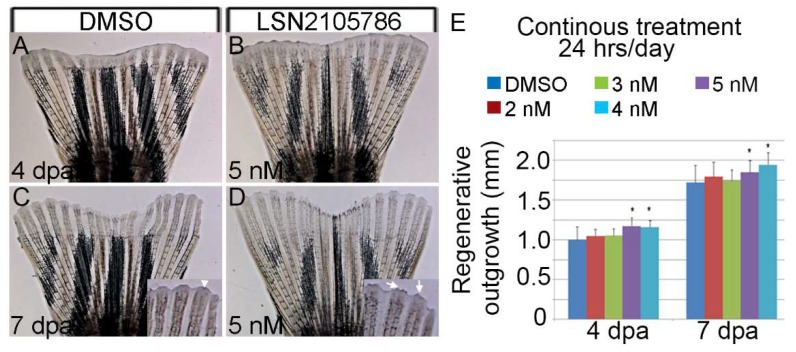
LSN 2105786 treatment accelerates fin regeneration. (**A**–**D**) Fin rays showing regeneration after DMSO (**A**,**C**) and LSN 2105786 treatment (**B**,**D**) at 4 (**A**,**B**) and 7 dpa (**C**,**D**). Insets show the mature fin tips in the LSN 2105786 treated fins (arrows) compared to DMSO treated fins (arrow head). (**E**) Graphs shows that continuous treatment of 4 and 5 nM LSN 2105786 produces increased regenerate lengths at 4 and 7 dpa compared to DMSO treatment. *, *p* < 0.05.

The optimal dosing regimen for maximal regenerative outgrowth was ascertained when caudal fin was amputated in fish that were treated continuously with various concentrations of LSN 2105786 (0.3, 1.0, 2.0, 3.0, 4.0, 5.0, 50.0 nM). Treatments using 4 or 5 nM LSN 2105786 were most effective at stimulating regeneration. In both the 4 and 5 nM continuous treatment groups, fin regenerates were significantly longer than control fins at 4 and 7 dpa (Figure 3A–D). Control regenerates were an average length of 1.00 mm at 4 dpa compared to 1.18 and 1.16 mm for the 4 and 5 nM treatment groups, respectively (Figure 2). This effect was evident at 7 dpa, showing regenerate average length of 1.72 mm in the control group and 1.85 and 1.95 mm, respectively, in the 4 and 5 nM treatment groups (Figure 3E).

### 3.4. LSN 2105786 Treatment Increased Expression of Wnt Targets and Downstream Genes in Regenerating Fin Tissue

**Figure 4:**
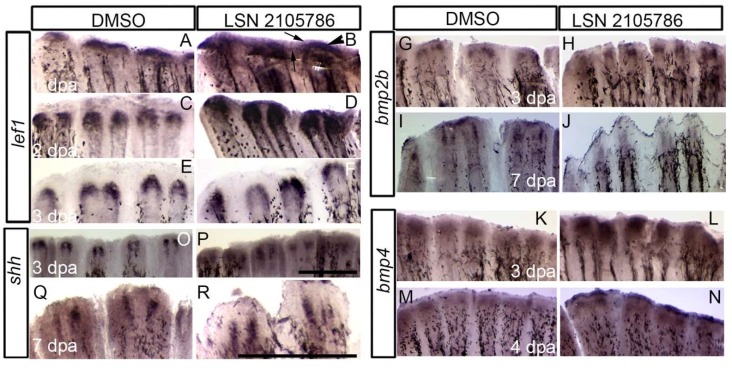
LSN 2105786 treatment increased expression of Wnt targets and downstream genes in regenerating fin tissue. (**A**–**F**) In situ hybridization detecting *lef1* transcript showed that the expression was restricted to the area in (arrowhead) and immediately surrounding (arrow) the blastema in DMSO treated fins, whereas, 5 nM LSN 2105786 treated fins showed an expansion of the expression domain as well as intensified staining at 1 and 2 dpa (**A**–**D**). Staining intensity at 3 dpa was not significantly altered in 5 nM LSN 2105786 treated fins compared to DMSO treated fins, but the expression area was expanded distally in treated fins (**E**,**F**). (**O**–**R**) In situ hybridization detecting *shh* transcript showed the expression in two distinct subsets of cells at the distal tip of the DMSO treated fins at 3 and 7 dpa (**O**,**Q**). Fins treated continuously with 5 nM LSN 2105786 showed expansion of the expression domain (P,R). (**G**–**J**) The expression of *bmp2b* was similar in the DMSO treated and 5 nM LSN 2105786 treated fins at 3 and 4 dpa. (**K**–**N**) In situ hybridization detecting *bmp4* transcript showed that the expression was dramatically increased in the 5 nM LSN 2105786 treated fin at 3 dpa (**L**) compared to DMSO treated fin (**K**). At 4 dpa, *bmp4* was robustly expressed in the distal most epidermis of LSN 2105786 treated fin (**N**) but was not detected in the same region of DMSO treated fins (**M**). Scale bars in panels P and R are 3 cm. Bar in P applies for all panels, except Q and R. Bar in R applies to Q and R.

To determine the spatial and temporal effects of GSK3β inhibition on Wnt responsive gene expression in fin regenerates, the Wnt/β-catenin signaling target gene, *lef1*, and downstream genes *shh*, *bmp2b*, and *bmp4* were analyzed by using in situ hybridization. Previous studies showed that *lef1* controls blastema formation [23,32], and it is expressed in the basal epidermal layer surrounding the blastema at 1 dpa. During the regenerative outgrowth phase (48 hpa and beyond), *lef1* is expressed in the distal blastema in two sets of cells on each side of the fin ray [23]. LSN 2105786 treated fin showed strongly expanded and more intense *lef1* expression at 1 and 2 dpa compared to vehicle treated control fins (Figure 4A–D). At 1 and 2 dpa, *lef1* was restricted to the area immediately surrounding the blastema in DMSO treated fins; whereas, LSN 2105786 treated fins showed an expansion of *lef1* expression in to the wound epidermis and inter ray tissue. At 3 dpa, *lef1* staining in two groups was similar (Figure 4E,F).

Previous fin regeneration studies showed that *bmp2b* and *bmp4* are expressed in the blastema [33,34]. During regeneration, *bmp2b* is expressed in differentiating scleroblasts and in the basal layer of the epidermis [33], and *bmp4* is expressed in the distal blastema [34]. The expression of *bmp2b* in the LSN 2105786 treated group was nearly indistinguishable from DMSO treated control group at 3 and 7 dpa (Figure 4G–J). In contrast, *bmp4* expression at the blastema region in 3 and 4 dpa fins showed weak *bmp4* expression at the blastema in controls, but LSN 2105786 treated group displayed more intense expression of *bmp4* (Figure 4K,L). At 4 dpa, *bmp4* expression was similarly expressed in the blastema, comparing in LSN 2105786 and DMSO treated control groups (Figure 4M,N), but higher expression in distal most epidermis was detected. The developmental signaling molecule *shh*, which is involved in patterning of various structures during vertebrate development, was previously shown to be expressed in the basal layer of wound epidermis in the regenerative outgrowth [33,35]. Similar to *lef1*, *shh* expression is typically restricted to two sets of cells on each side of the distal tip of the fin ray during the regenerative outgrowth phase. In the LSN 2105786 treated fins, there was a slight expansion of the *shh* expression domain at 3 dpa, with *shh* transcripts being detected in the tissue proximal to the blastema and extending to near the amputation plane (Figure 4O,P). At 4 dpa, the intensity of *shh* staining remained high in the LSN 2105786 treated group as compared to the DMSO treated control group. By 7 dpa, *shh* expression was similar in LSN 2105786 and DMSO treated control groups (Figure 4Q,R).

### 3.5. LSN 2105786 Increases Cell Proliferation in Regenerating Fins

**Figure 5:**
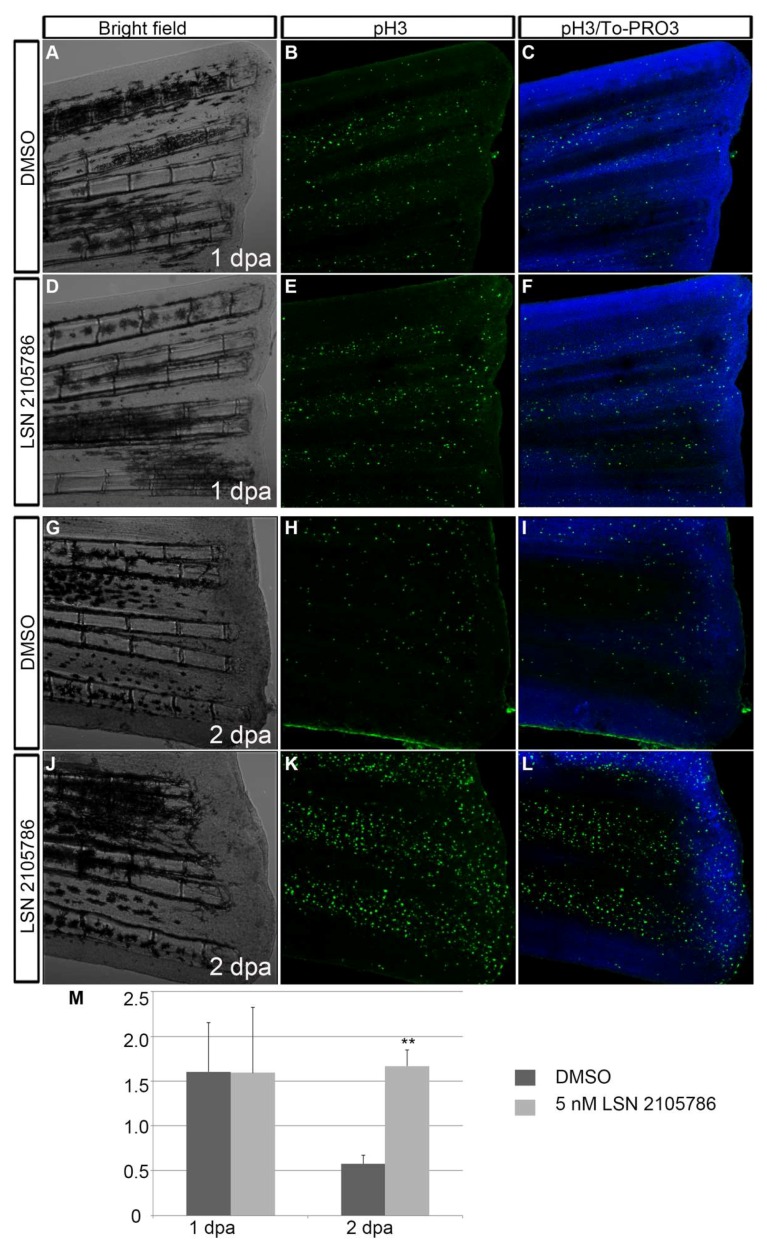
LSN 2105786 treatment increases cell proliferation in regenerating fins. (**A**–**L**) Phosphohistone 3 immunostaining showed similar labeling of proliferated pH3 positive cells in the DMSO treated (**B**,**C**) and 5 nM LSN 2105786 treated fins at 1 dpa (**E**,**F**), but at 2 dpa, 5 nM LSN 2105786 treated fins (**K**,**L**) showed dramatic increase of pH3 positive cells compared to DMSO treated fins (**H**,**I**). (**M**) Graph shows the number of pH3 positive cells at 1 and 2 dpa. **, *p* < 0.001.

The Wnt signaling pathway controls cell proliferation throughout development [36]. Continuous LSN 2105786 treatment was tested in the regenerating fin tissue, and cell proliferation was examined. Phosphorylated histone-3, a marker for chromatin condensation during M phase, was used examine proliferation in the 1 and 2 dpa regenerating fin. Proliferating cells were counted, and the number of nuclei per unit area was calculated. Numbers of proliferating cells at 1 dpa was nearly identical in treated and control fins (Figure 5A–F,M), but at 2 dpa, there was an approximately three-fold increase in numbers of proliferating cells in LSN 2105786 treated fins as compared with controls (Figure 5G–M).

### 3.6. LSN 2105786 Treatment Increases Osteoblast Differentiation

**Figure 6:**
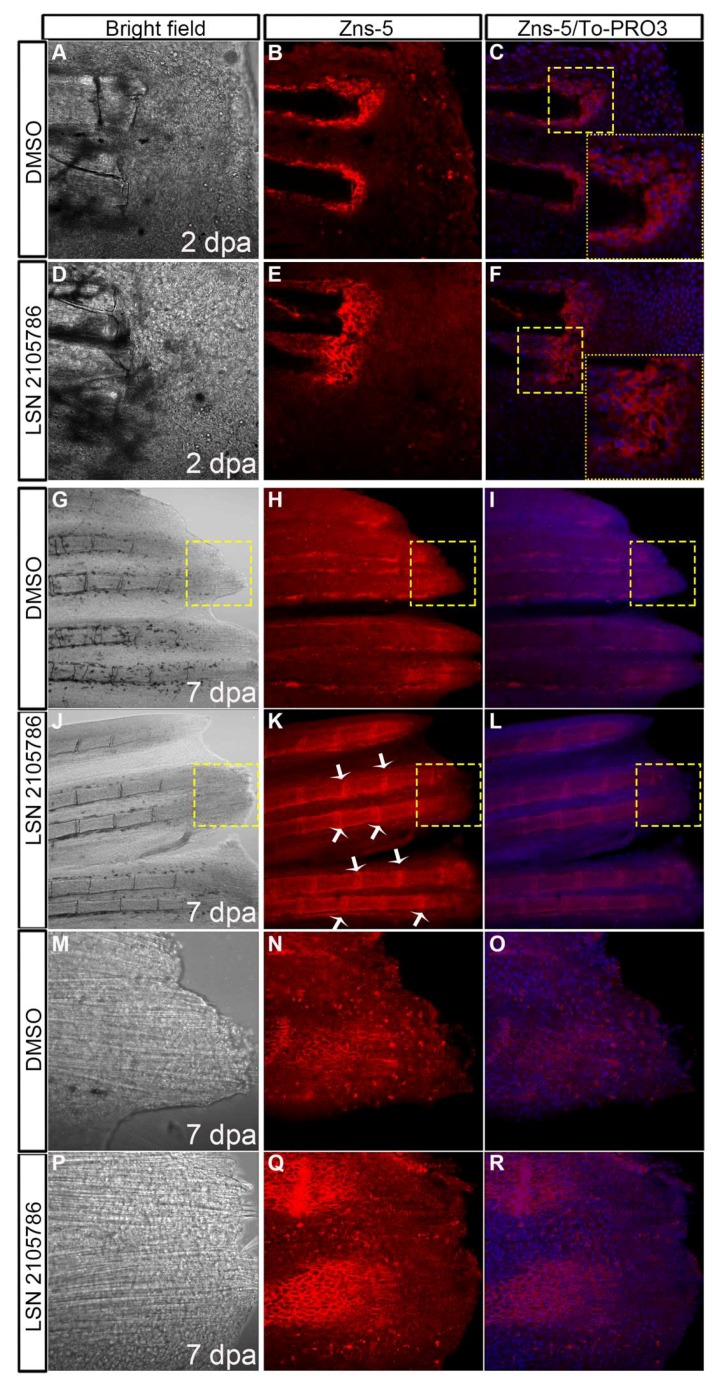
LSN 2105786 treatment increases osteoblast differentiation. (**A**–**F**) Zns-5 antibody staining showed an expansion of the labeling area in the blastema region in 5 nM LSN 2105786 treated fins (**E**,**F**) compared to DMSO treated fins (**B**,**C**) at 2 dpa. Yellow boxed areas are shown in high magnification. (**G**–**L**) Zns-5 antibody staining of 7 dpa fins showed Zns-5 labeling in the lateral regions of the hemirays of DMSO treated fish (**H**,**I**), but 5 nM LSN 2105786 treated fish exhibited Zns-5 positive cells in the lateral regions of the hemirays and in the joints between the segments (**K**,**L**). Arrows point to the joints. (**M**–**R**) High magnification images of the boxed areas are shown in G–L. The 5 nM LSN 2105786 treated fins show Zns-5 labeling in the distal region of the bony rays (**N**,**O**) compared to DMSO treated fins (**Q**,**R**).

To test the effect of GSK3β inhibitor LSN 2105786 on osteoblasts, fins were immunostained with the monoclonal antibody Zns-5, which labels differentiated osteoblasts and nuclear stain To-PRO3 iodide. Confocal microscopy images of fins treated with 5 nM LSN 2105786 showed larger Zns-5 positive area in the blastema compared to DMSO treated fins at 2 dpa (Figure 6A–F). At 7 dpa, regenerated bony rays of 5 nM LSN 2105786 treated fins were strongly stained with Zns-5 antibody. Zns-5 positive osteoblasts were distributed in the lateral regions of the hemirays of DMSO treated fish, but 5 nM LSN 2105786 treated fish exhibited Zns-5 positive osteoblasts in the lateral regions of the hemirays as well as in the joints between the segments (Figure 6G–L). High magnification images showed more Zns-5 staining in the distal region of the bony rays of 5 nM LSN 2105786 treated fish compared to DMSO treated fish (Figure 6M–R).

## 4. Discussion

Previously, Flemming et al. [37] and others showed that the small size, ease of care and relatively low cost of zebrafish are important advantages to using fish for bone research and drug discovery screening [38,39]. Zebrafish afford the important opportunity to ascertain quickly efficacious doses of compounds on target tissues, optimal dosing regimens and toxicity of novel synthetic molecules, which may or may not be off target. Additionally, caudal fin regeneration studies allow the generation of data on bone healing mechanisms, particularly on the earliest responses to injury.

Our studies are in agreement with Stoick-Cooper et al., showing that activating Wnt/β-catenin signaling improves zebrafish caudal fin regenerative outgrowth [22]. LSN 2105786 treatment increased regenerate length at 7 dpa by 13% as compared to controls. Additionally, previous studies showed that increased Wnt/β-catenin signaling in heterozygous *axin1*^+/-^ fish had increased in regenerative outgrowth, as compared to wild type fish at the same time point [25]. Interestingly, intermittent LSN 2105786 treatment was less effective than continuous treatment, which is in agreement with previously published data showing that pulses of a *wnt8* transgene expression was sufficient to increase cell proliferation, but did not affect the length of fin regenerates [25]. A possible limitation of this study is that zebrafish can regenerate fins normally, without intervention. So, the extent to which regenerative outgrowth can be augmented in healthy fish may be limited. Therefore, in future studies, it may be useful to test GSK3β inhibitor and other compounds that promote regeneration in defective fish with impaired regenerative processes, or with abnormal bone phenotypes, perhaps due to aging or chronic alcohol exposure.

The inhibitory effects on regeneration observed following continuous LSN 2105786 treatment at higher concentrations above 5 mM may indicate that over-activation of the Wnt/β-catenin pathway excessively stimulates differentiation programs, perhaps depleting the progenitor cell population and preventing outgrowth. Additional studies aimed at determining effects of higher concentrations of GSK3β inhibitor on Wnt/β-catenin signaling in fin regeneration are needed, and these studies may provide insight into modulation of the regeneration process.

GSK3β inhibitor effects recapitulated previous studies using ectopic Wnt signaling phenotypes in embryos, showing LSN 2105786 acts specifically. For example, upregulation of the direct Wnt target, *lef1* provides strong evidence that LSN 2105786 activates canonical Wnt signaling during fin regeneration. Experiments using human and zebrafish GSK3β synthetic mRNA to rescue Gsk3β morpholino oligonucleotide knockdown phenotypes also strongly supports the conclusion that the human and zebrafish enzyme are interchangeable in the zebrafish embryo. Together, these results indicate that the zebrafish is a useful model to study GSK3β effects on Wnt/β-catenin signaling pathways.

LSN 2105786 treatment was sufficient to activate cell proliferation and expression of downstream genes in Wnt/β-catenin signaling. LSN 2105786 treatment, which induces blastema cell proliferation and outgrowth, may help induce and maintain cell proliferation and regenerative outgrowth. Expression of *shh* was expanded at 3 dpa, which induces proliferation and alignment of osteoblasts in the regenerating fin [35].

In summary, our analysis of the effects of GSK3β inhibition on zebrafish fin regeneration showed that enhanced Wnt/β-catenin signaling produced Wnt/β-catenin target gene expression; cell proliferation; and more rapid fin regeneration. Taken together, the results indicate that chemical biology of zebrafish fin regeneration will help us understand Wnt-mediated bone growth and repair. In particular, mechanistic features can be studied and applied to clinically important questions for potential bone healing therapies.

## References

[1] Baron R., Kneissel M. (2013). WNT signaling in bone homeostasis and disease: From human mutations to treatments. Nat. Med..

[2] Moon R.T., Bowerman B., Boutros M., Perrimon N. (2002). The promise and perils of Wnt signaling through beta-catenin. Science.

[3] Agholme F., Aspenberg P. (2011). Wnt signaling and orthopedics, an overview. Acta Orthopaed..

[4] Westendorf J.J., Kahler R.A., Schroeder T.M. (2004). Wnt signaling in osteoblasts and bone diseases. Gene.

[5] Gong Y., Slee R.B., Fukai N., Rawadi G., Roman-Roman S., Reginato A.M., Wang H., Cundy T., Glorieux F.H., Lev D. (2001). Osteoporosis-Pseudoglioma Syndrome Collaborative, G. LDL receptor-related protein 5 (LRP5) affects bone accrual and eye development. Cell.

[6] Boyden L.M., Mao J., Belsky J., Mitzner L., Farhi A., Mitnick M.A., Wu D., Insogna K., Lifton R.P. (2002). High bone density due to a mutation in LDL-receptor-related protein 5. N. Engl. J. Med..

[7] Holmen S.L., Zylstra C.R., Mukherjee A., Sigler R.E., Faugere M.C., Bouxsein M.L., Deng L., Clemens T.L., Williams B.O. (2005). Essential role of beta-catenin in postnatal bone acquisition. J. Biol. Chem..

[8] Spencer G.J., Utting J.C., Etheridge S.L., Arnett T.R., Genever P.G. (2006). Wnt signalling in osteoblasts regulates expression of the receptor activator of NFkappaB ligand and inhibits osteoclastogenesis in vitro. J. Cell Sci..

[9] Hu H., Hilton M.J., Tu X., Yu K., Ornitz D.M., Long F. (2005). Sequential roles of Hedgehog and Wnt signaling in osteoblast development. Development.

[10] Hill T.P., Spater D., Taketo M.M., Birchmeier W., Hartmann C. (2005). Canonical Wnt/beta-catenin signaling prevents osteoblasts from differentiating into chondrocytes. Dev. Cell.

[11] Day T.F., Guo X., Garrett-Beal L., Yang Y. (2005). Wnt/beta-catenin signaling in mesenchymal progenitors controls osteoblast and chondrocyte differentiation during vertebrate skeletogenesis. Dev. Cell.

[12] Bennett C.N., Ouyang H., Ma Y.L., Zeng Q., Gerin I., Sousa K.M., Lane T.F., Krishnan V., Hankenson K.D., MacDougald O.A. (2007). Wnt10b increases postnatal bone formation by enhancing osteoblast differentiation. J. Bone Min. Res..

[13] Zhong N., Gersch R.P., Hadjiargyrou M. (2006). Wnt signaling activation during bone regeneration and the role of Dishevelled in chondrocyte proliferation and differentiation. Bone.

[14] Chen Y., Whetstone H.C., Lin A.C., Nadesan P., Wei Q., Poon R., Alman B.A. (2007). Beta-catenin signaling plays a disparate role in different phases of fracture repair: Implications for therapy to improve bone healing. PLoS Med..

[15] Li X., Grisanti M., Fan W., Asuncion F.J., Tan H.L., Dwyer D., Han C.Y., Yu L., Lee J., Lee E. (2011). Dickkopf-1 regulates bone formation in young growing rodents and upon traumatic injury. J. Bone Min. Res..

[16] Ominsky M.S., Li C., Li X., Tan H.L., Lee E., Barrero M., Asuncion F.J., Dwyer D., Han C.Y., Vlasseros F. (2011). Inhibition of sclerostin by monoclonal antibody enhances bone healing and improves bone density and strength of nonfractured bones. J. Bone Min. Res..

[17] Florio M., Gunasekaran K., Stolina M., Li X., Liu L., Tipton B., Salimi-Moosavi H., Asuncion F.J., Li C., Sun B. (2016). A bispecific antibody targeting sclerostin and DKK-1 promotes bone mass accrual and fracture repair. Nat. Commun..

[18] Kulkarni N.H., Halladay D.L., Miles R.R., Gilbert L.M., Frolik C.A., Galvin R.J., Martin T.J., Gillespie M.T., Onyia J.E. (2005). Effects of parathyroid hormone on Wnt signaling pathway in bone. J. Cell. Biochem..

[19] Alkhiary Y.M., Gerstenfeld L.C., Krall E., Westmore M., Sato M., Mitlak B.H., Einhorn T.A. (2005). Enhancement of experimental fracture-healing by systemic administration of recombinant human parathyroid hormone (PTH 1-34). J. Bone Joint Surg..

[20] Aspenberg P., Genant H.K., Johansson T., Nino A.J., See K., Krohn K., Garcia-Hernandez P.A., Recknor C.P., Einhorn T.A., Dalsky G.P. (2010). Teriparatide for acceleration of fracture repair in humans: A prospective, randomized, double-blind study of 102 postmenopausal women with distal radial fractures. J. Bone Min. Res..

[21] Aspenberg P., Johansson T. (2010). Teriparatide improves early callus formation in distal radial fractures. Acta Orthopaed..

[22] Stoick-Cooper C.L., Moon R.T., Weidinger G. (2007). Advances in signaling in vertebrate regeneration as a prelude to regenerative medicine. Genes Dev..

[23] Poss K.D., Shen J., Keating M.T. (2000). Induction of lef1 during zebrafish fin regeneration. Dev. Dyn..

[24] Kawakami Y., Rodriguez Esteban C., Raya M., Kawakami H., Marti M., Dubova I., Izpisua Belmonte J.C. (2006). Wnt/beta-catenin signaling regulates vertebrate limb regeneration. Genes Dev..

[25] Stoick-Cooper C.L., Weidinger G., Riehle K.J., Hubbert C., Major M.B., Fausto N., Moon R.T. (2007). Distinct Wnt signaling pathways have opposing roles in appendage regeneration. Development.

[26] Kulkarni N.H., Onyia J.E., Zeng Q., Tian X., Liu M., Halladay D.L., Frolik C.A., Engler T., Wei T., Kriauciunas A. (2006). Orally bioavailable GSK-3alpha/beta dual inhibitor increases markers of cellular differentiation in vitro and bone mass in vivo. J. Bone Min. Res..

[27] Dorsky R.I., Sheldahl L.C., Moon R.T. (2002). A transgenic Lef1/beta-catenin-dependent reporter is expressed in spatially restricted domains throughout zebrafish development. Dev. Biol..

[28] Westerfield M., Eugene O.R. (2000). The Zebrafish Book.

[29] Kelley L.A., Mezulis S., Yates C.M., Wass M.N., Sternberg M.J. (2015). The Phyre2 web portal for protein modeling, prediction and analysis. Nat. Protoc..

[30] Lee H.C., Tsai J.N., Liao P.Y., Tsai W.Y., Lin K.Y., Chuang C.C., Sun C.K., Chang W.C., Tsai H.J. (2007). Glycogen synthase kinase 3 alpha and 3 beta have distinct functions during cardiogenesis of zebrafish embryo. BMC Dev. Biol..

[31] Van de Water S., van de Wetering M., Joore J., Esseling J., Bink R., Clevers H., Zivkovic D. (2001). Ectopic Wnt signal determines the eyeless phenotype of zebrafish masterblind mutant. Development.

[32] Blum N., Begemann G. (2012). Retinoic acid signaling controls the formation, proliferation and survival of the blastema during adult zebrafish fin regeneration. Development.

[33] Poss K.D., Keating M.T., Nechiporuk A. (2003). Tales of regeneration in zebrafish. Dev. Dyn..

[34] Murciano C., Fernandez T.D., Duran I., Maseda D., Ruiz-Sanchez J., Becerra J., Akimenko M.A., Mari-Beffa M. (2002). Ray-interray interactions during fin regeneration of Danio rerio. Dev. Biol..

[35] Quint E., Smith A., Avaron F., Laforest L., Miles J., Gaffield W., Akimenko M.A. (2002). Bone patterning is altered in the regenerating zebrafish caudal fin after ectopic expression of sonic hedgehog and bmp2b or exposure to cyclopamine. Proc. Natl. Acad. Sci. USA.

[36] Willert K., Jones K.A. (2006). Wnt signaling: Is the party in the nucleus?. Genes Dev..

[37] Fleming A., Sato M., Goldsmith P. (2005). High-throughput in vivo screening for bone anabolic compounds with zebrafish. J. Biomol. Screen..

[38] Sarmah S., Marrs J.A. (2016). Zebrafish as a Vertebrate Model System to Evaluate Effects of Environmental Toxicants on Cardiac Development and Function. Int. J. Mol. Sci..

[39] Asnani A., Peterson R.T. (2014). The zebrafish as a tool to identify novel therapies for human cardiovascular disease. Dis. Models Mech..

